# Supplementary material for: Inducible mechanisms of disease tolerance provide an alternative strategy of acquired immunity to malaria
Source: eLife. 2021 Mar 23;10:e63838. doi: 10.7554/eLife.63838 (PMC7987336; doi:10.7554/eLife.63838)
Supplement: Supplementary file 1. — Flow cytometry was performed using the listed antibodies and panels. We gated myeloid cells and their progenitors in bone marrow, blood, and spleen using FlowJo v9; flow profiles of uninfected mice are displayed alongside the acute phase of a first malaria episode (P. chabaudi AJ). In every case, gating was performed identically between uninfected and infected mice with one exception (marked with an asterisk); to identify myeloid and erythroid progenitors in the bone marrow of infected mice we had to adjust our first gate (on lineage negative live singlets) due to the well-known upregulation of Sca-1 during acute infection (Belyaev et al., 2010). Note that CD115 (Csf1r) was replaced with CD11c when sorting monocytes as engagement of the Csf1 receptor has been shown to induce transcriptional changes (Jung et al., 2000). [file elife-63838-supp1.pdf]

Supplementary file 1

progenitors [bone marrow & spleen]	
BV421	**CD71**
BV605	**Sca1**
FITC	**CD34**
PerCP EF710	**cKit**
PE	**CD27**
PE Cy7	***CD16/32****
APC [lineage]	**CD3**<br>**CD4**<br>**CD19**<br>**NK1.1**<br>**Ter119**<br>**CD8a**<br>**Ly6G**<br>**CD11c**<br>**CD11b**

inflammatory monocytes [bone marrow]	
BV421	**Ly6C**
BV605	**CD115**
FITC	**Ly6G**
PerCP EF710	**c Kit**
PE	**CD135**
PE Cy7	**CD11b**
lineage [APC]	**CD3**<br>**CD4**<br>**CD19**<br>**NK1.1**<br>**Ter119**<br>**CD8a**<br>**CD11c**

inflammatory monocytes [blood & spleen]	
BV421	**Ly6C**
BV605	**CD115**
FITC	**IAb**
PerCP EF710	**Ly6G**
PE	**CD11c**
PE Cy7	**CD11b**
APC [lineage]	**CD3**<br>**CD4**<br>**CD19**<br>**NK1.1**<br>**Ter119**

red pulp Mϕ [spleen]	
BV421	**F4/80**
BV605	**CD11b**
FITC	*auto***
PerCP EF710	**B220**
PE	**CD11c**
APC [lineage]	**CD3**<br>**CD4**<br>**CD19**<br>**NK1.1**<br>**Ter119**<br>**Ly6G**

iron recycling Mϕ [bone marrow]	
BV421	**F4/80**
BV605	**CD115**
FITC	**CD11b**
PerCP Cy5.5	**VCAM-1**
PE	**CD11c**
PE Cy7	**CD169**
APC [lineage]	**CD3**<br>**CD4**<br>**CD19**<br>**NK1.1**<br>**Ter119**<br>**Ly6G**

patrolling monocytes [blood]	
BV421	**Ly6C**
BV605	**CD115**
FITC	**IAb**
PerCP Cy5.5	**Cx3cr1**
PE	***Nr4a1*****
PE Cy7	**CD11b**
APC [lineage]	**CD3**<br>**CD4**<br>**CD19**<br>**NK1.1**<br>**Ter119**<br>**CD8a**<br>**Ly6G**

antibody details		
**anti-mouse**	**clone**	**supplier**
**B220**	RA3-6B2	ebioscience
**CD3ε**	145-2C11	biolegend
**CD4**	RM4-5	biolegend
**CD8a**	53-6.7	biolegend
**CD11b**	M1/70	biolegend
**CD11c**	N418	biolegend
**CD16/32**	93	ebioscience
**CD19**	6D5	biolegend
**CD27**	LG.7F9	ebioscience
**CD34**	RAM34	ebioscience
**CD71**	RI7217	biolegend
**CD115 = Csf1r**	AFS98	biolegend
**CD135 = Flt3**	A2F10	ebioscience
**CD169**	3D6.112	biolegend
**cKit = CD117**	2B8	ebioscience
**Cx3cr1**	SA011F11	biolegend
**F4/80**	BM8	biolegend
**IAb**	AF6-120.1	biolegend
**Ly6C**	HK1.4	biolegend
**Ly6G**	1A8-Ly6g	ebioscience
**NK1.1**	PK136	biolegend
**Nr4a1 = Nur77**	12.14	ebioscience
**Sca1 = Ly6A/E**	D7	biolegend
**Ter119**	Ter119	biolegend
**VCAM-1**	429	biolegend

Mϕ macrophages
* do not use TruStain FcX
** autofluorescence
*** intracellular stain

Supplementary File 1 - continued next page

[blood & spleen] **monocytes** & **neutrophils**

uninfected

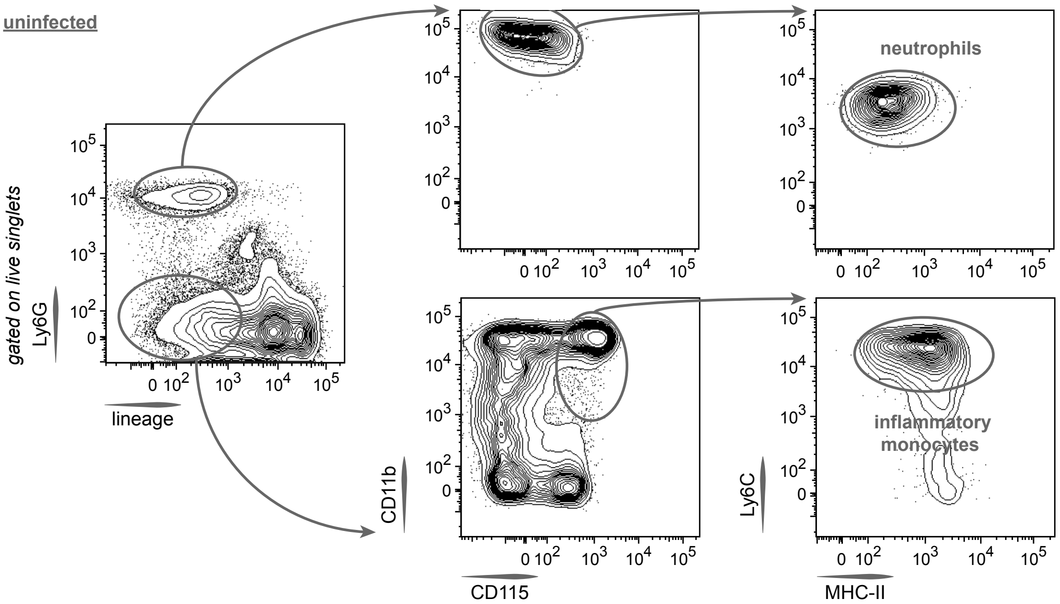

1st infection - acute

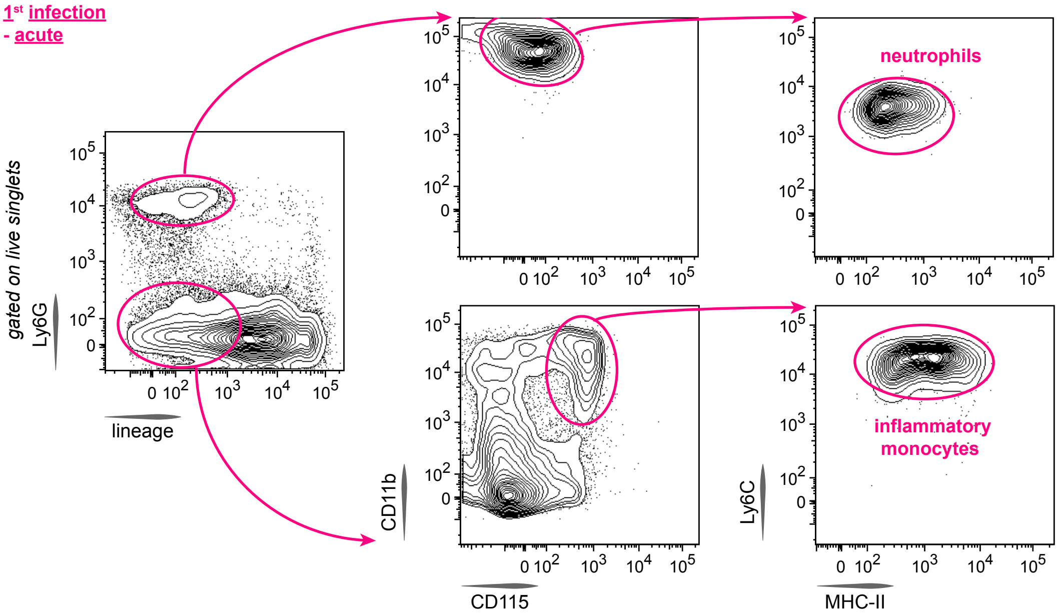

Supplementary File 1 - continued next page

[bone marrow] **monocytes** & **neutrophils**

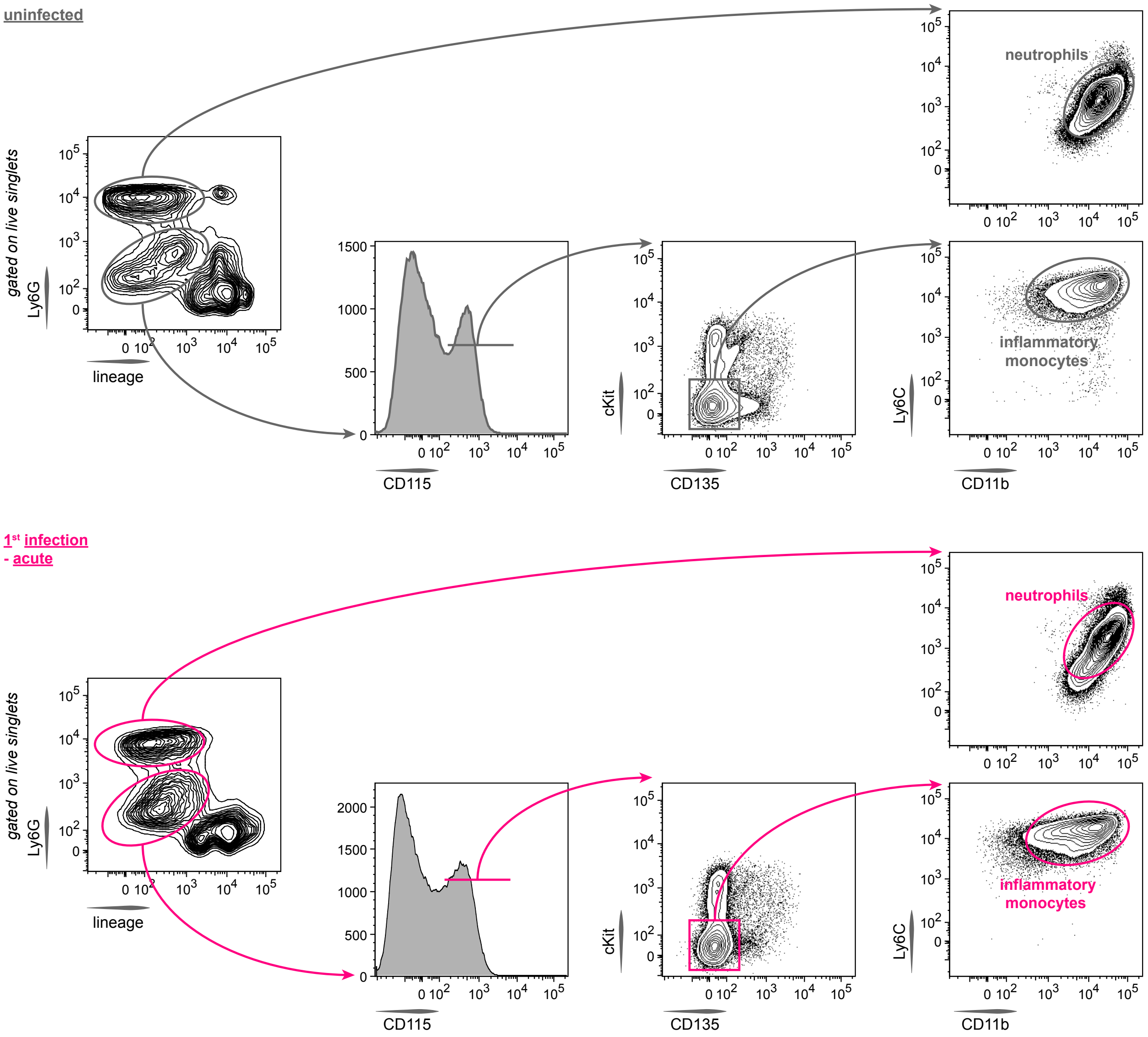

Supplementary File 1 - continued next page

[bone marrow] **iron recycling macrophages (Mϕ)**

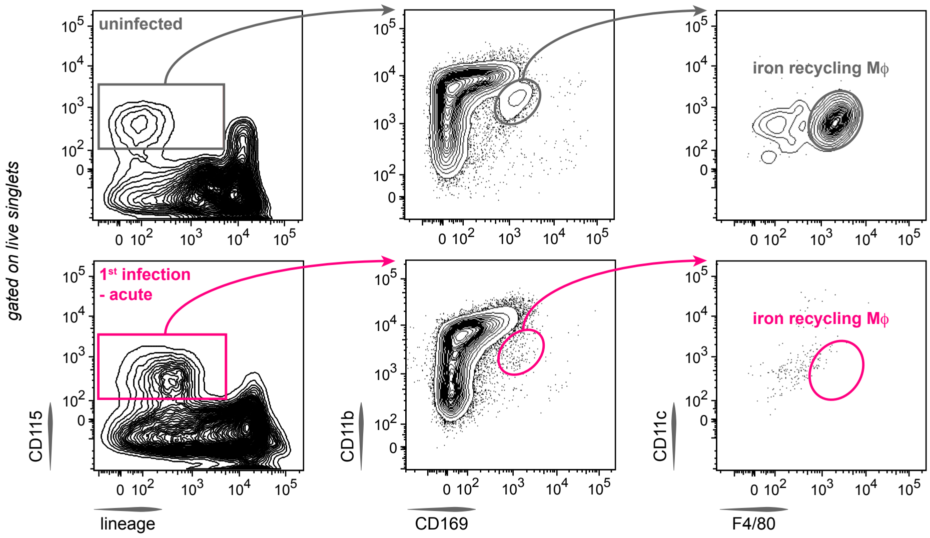

[blood] **patrolling monocytes**

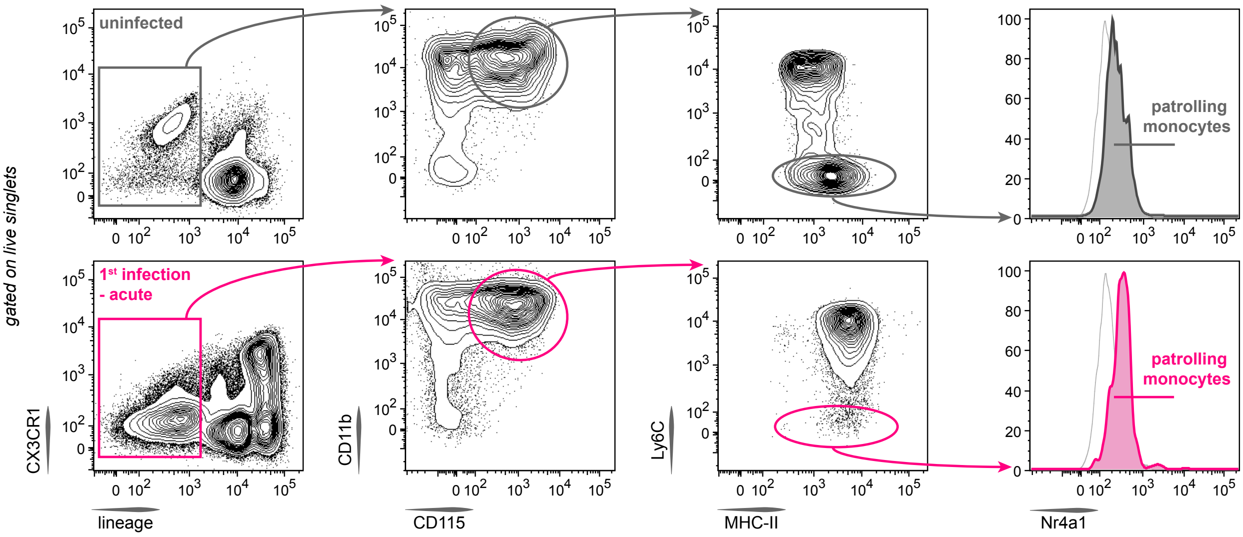

[spleen] **red pulp macrophages (Mϕ)**

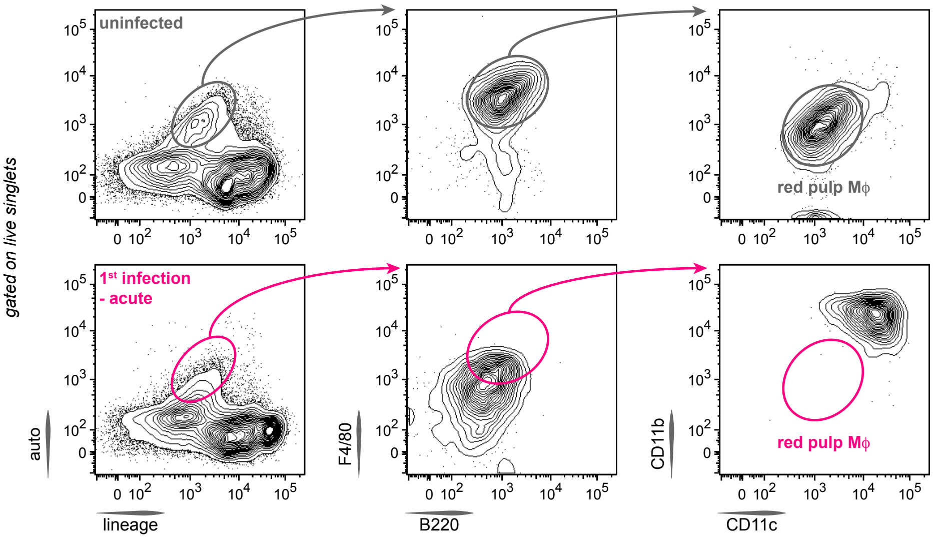

Supplementary File 1 - continued next page

[bone marrow & spleen] **myeloid** & **erythroid progenitors**

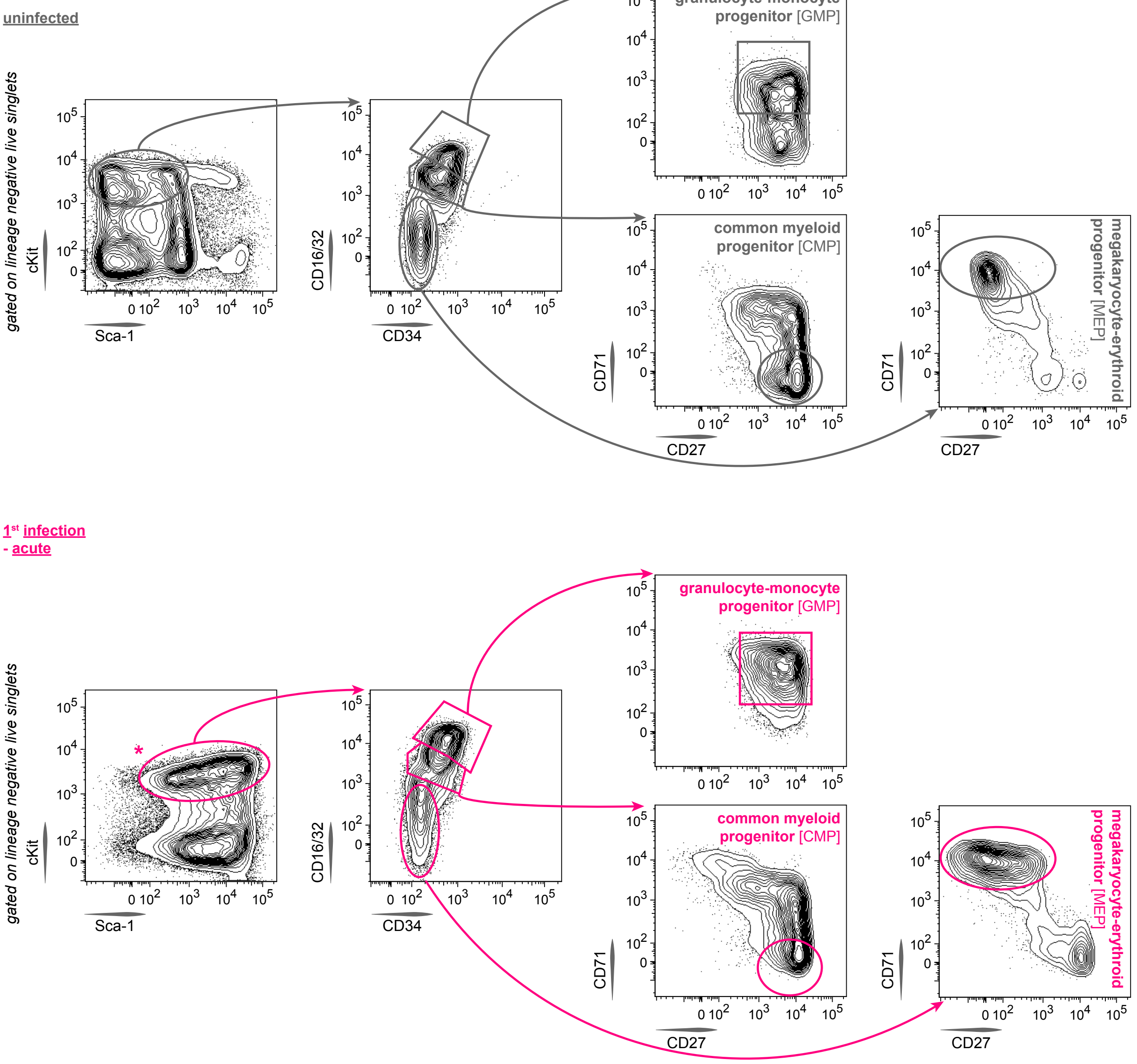

Supplementary File 1
