# Supplementary material for: Inducible mechanisms of disease tolerance provide an alternative strategy of acquired immunity to malaria
Source: eLife. 2021 Mar 23;10:e63838. doi: 10.7554/eLife.63838 (PMC7987336; doi:10.7554/eLife.63838)
Supplement: Transparent reporting form [file elife-63838-transrepform.docx]

***eLife’s* transparent reporting form**

We encourage authors to provide detailed information *within their submission* to facilitate the interpretation and replication of experiments. Authors can upload supporting documentation to indicate the use of appropriate reporting guidelines for health-related research (see [EQUATOR Network](http://www.equator-network.org/%20)), life science research (see the [BioSharing Information Resource](https://biosharing.org/)), or the [ARRIVE guidelines](http://www.plosbiology.org/article/info:doi/10.1371/journal.pbio.1000412) for reporting work involving animal research. Where applicable, authors should refer to any relevant reporting standards documents in this form.

If you have any questions, please consult our Journal Policies and/or contact us: [editorial@elifesciences.org](mailto:editorial@elifesciences.org).

**Sample-size estimation**

- You should state whether an appropriate sample size was computed when the study was being designed
- You should state the statistical method of sample size computation and any required assumptions
- If no explicit power analysis was used, you should describe how you decided what sample (replicate) size (number) to use

Please outline where this information can be found within the submission (e.g., sections or figure legends), or explain why this information doesn’t apply to your submission:

Prior to undertaking this study power calculations were performed by the Centre for Infection, Immunity and Evolution at the University of Edinburgh. Data were available from our own prior studies exploring mosquito transmission of *Plasmodium chabaudi* [Spence *et al*, **Nature** (2013)]. Sample size was calculated using G*power and for all variables the power was set to 0.80 and alpha to 0.05. As one example, it was estimated that a sample size of 12 mice per group should be sufficient to detect a difference in disease severity (e.g. anaemia) with 80% power and 95% confidence.

**Replicates**

- You should report how often each experiment was performed
- You should include a definition of biological versus technical replication
- The data obtained should be provided and sufficient information should be provided to indicate the number of independent biological and/or technical replicates
- If you encountered any outliers, you should describe how these were handled
- Criteria for exclusion/inclusion of data should be clearly stated
- High-throughput sequence data should be uploaded before submission, with a private link for reviewers provided (these are available from both GEO and ArrayExpress)

Please outline where this information can be found within the submission (e.g., sections or figure legends), or explain why this information doesn’t apply to your submission:

At least 3 independent experiments were performed for phenotyping the course and outcome of infection and rechallenge (parasitaemia, anaemia, hypothermia and sequestration). At least 2 independent experiments were performed for flow cytometry, histology and ELISA (inc. inflammation and endothelium activation). RNA-sequencing and microarray experiments were carried out once but sample sizes were calculated (as described above) to ensure these experiments were sufficiently powered to detect differences in gene expression between groups. And ChIPseq data derive from 3 independent experiments.

Each sample represents an individual mouse within an experimental group (a biological replicate) and sample sizes are given for every experiment in the Figure legends. For ChIPseq data each replicate represents two pooled mice within an experimental group; pooling was required to obtain sufficient cell numbers to carry out immunoprecipitation using three different antibodies (H3K27ac, H3K4me1, H3K9me3) on the same biological sample. Only replicates that surpassed a high IP efficiency threshold were taken forward for downstream analyses; details of these thresholds and the number of replicates that passed this QC step in each experimental group are given in the Methods. Note that a detailed step-by-step protocol of our optimised low input ChIPseq methodology is available at protocols.io ([dx.doi.org/10.17504/protocols.io.bja3kign](https://dx.doi.org/10.17504/protocols.io.bja3kign)). Technical replicates were not included in this study.

Data were excluded in two specific circumstances - first, when chronic infection could not be confirmed by qPCR (limit of detection 5 parasites*μl^-1^) then these mice were excluded from the study. And second, when IP efficiency did not surpass the required threshold in ChIPseq experiments (see Methods) then these replicates were excluded from downstream analyses. Outliers were not encountered in phenotyping experiments or immunological analyses; nevertheless, outlier detection was ON in DESeq2 when calling differentially expressed genes to improve model stability and sensitivity.

All RNAseq, ChIPseq and microarray datasets are published and are accessible through GEO SuperSeries accession number GSE150479. These data can be found here:

<https://www.ncbi.nlm.nih.gov/geo/query/acc.cgi?acc=GSE150479>

**Statistical reporting**

- Statistical analysis methods should be described and justified
- Raw data should be presented in figures whenever informative to do so (typically when N per group is less than 10)
- For each experiment, you should identify the statistical tests used, exact values of N, definitions of center, methods of multiple test correction, and dispersion and precision measures (e.g., mean, median, SD, SEM, confidence intervals; and, for the major substantive results, a measure of effect size (e.g., Pearson's r, Cohen's d)
- Report exact p-values wherever possible alongside the summary statistics and 95% confidence intervals. These should be reported for all key questions and not only when the p-value is less than 0.05.

Please outline where this information can be found within the submission (e.g., sections or figure legends), or explain why this information doesn’t apply to your submission:

Details of the methodology used to call differentially expressed genes (RNA-sequencing and microarray) are provided in the Methods and Results. We also specify the sample number (N) for each pairwise comparison, the method used to adjust for multiple testing and the adjusted p value that we considered significant. ClueGO was used for functional gene enrichment analysis and in this case we provide details of all parameters applied (e.g. Bonferroni step down method of correction) in the Methods. The key details (inc. sample size and the adj p value considered to be significant) are also given in the Figure legends for ease of access.

The methodology used to call peaks, identify differentially modified regions and visualise ChIPseq data is given in detail in the Methods. This information includes the number of replicates in each experimental group, the origin of non-immunoprecipitated DNA used as input, the parameters used to call peaks for each histone modification, the IP efficiency threshold applied, the filtering of peaks to retain only those that are significant and the pooling of replicates to create tag directories for visualisation. Note that we adhered to the Reference Epigenome Standards as set out by the International Human Epigenome Consortium (http://ihec-epigenomes.org) throughout data collection and analysis.

For all other data types (e.g. parasitaemia, cell counts, plasma analytes etc.) we show data as median and IQR, and include max. and min. values in each group to highlight possible outliers. We also show each individual replicate when we feel that this additional information will help the reader assess the significance of a finding. Samples below the limit of detection (e.g. chronicity of infection) are clearly labelled. All of these details are given throughout the Results, Figure legends and Methods.

(For large datasets, or papers with a very large number of statistical tests, you may upload a single table file with tests, Ns, etc., with reference to sections in the manuscript.)

**Group allocation**

- Indicate how samples were allocated into experimental groups (in the case of clinical studies, please specify allocation to treatment method); if randomization was used, please also state if restricted randomization was applied
- Indicate if masking was used during group allocation, data collection and/or data analysis

Please outline where this information can be found within the submission (e.g., sections or figure legends), or explain why this information doesn’t apply to your submission:

Randomisation of mice to experimental groups was not included in the study design; nevertheless, every mouse enrolled in an experiment was genetically identical, age and sex-matched, obtained from a single breeding facility and acclimatised to a reversed 12-hour dark/light cycle at least 10-days prior to the start of experimental procedures. Researchers were not blinded during data collection or analysis.

**Additional data files (“source data”)**

- We encourage you to upload relevant additional data files, such as numerical data that are represented as a graph in a figure, or as a summary table
- Where provided, these should be in the most useful format, and they can be uploaded as “Source data” files linked to a main figure or table
- Include model definition files including the full list of parameters used
- Include code used for data analysis (e.g., R, MatLab)
- Avoid stating that data files are “available upon request”

Please indicate the figures or tables for which source data files have been provided:

Supplementary File 1 includes details of all antibodies and gating strategies used for flow cytometry and cell sorting (supports Figures 1 and 3, Figure 1 - figure supplement 2, Figure 2 - figure supplement 2 and Figure 3 - figure supplement 1).

Supplementary File 2 includes summary statistics for genes annotated with a differentially marked region (supports Figure 6 and Figure 6 - figure supplement 1).
